# Robust DOA Estimation via a Deep Learning Framework with Joint Spatial–Temporal Information Fusion

**DOI:** 10.3390/s25103142

**Published:** 2025-05-15

**Authors:** Yonghong Zhao, Xiumei Fan, Jisong Liu

**Affiliations:** 1 School of Automation and Information Engineering, Xi’an University of Technology, Xi’an 710048, China; zhaoyh2018@xaut.edu.cn (Y.Z.); 2230320112@stu.xaut.edu.cn (J.L.); 2 Shaanxi Key Laboratory of Complex System Control and Intelligent Information Processing, Xi’an University of Technology, Xi’an 710048, China

**Keywords:** direction-of-arrival estimation, deep learning, depthwise separable convolution, LSTM

## Abstract

In this paper, we propose a robust deep learning (DL)-based method for Direction-of-Arrival (DOA) estimation. Specifically, we develop a novel CRDCNN-LSTM network architecture, which integrates a Cross-Residual Depthwise Convolutional Neural Network (CRDCNN) with a Long Short-Term Memory (LSTM) module for effective capture of both spatial and temporal features. The CRDCNN employs multi-level cross-residual connections and depthwise separable convolutions to enhance feature diversity while mitigating issues such as gradient vanishing and overfitting. Furthermore, a customized FD loss function, combining Focal Loss and Dice Loss, is introduced to emphasize low-confidence samples and promote sparsity in the spatial spectrum, thereby improving the precision and overall effectiveness of DOA estimation. A post-processing strategy based on peak detection and quadratic interpolation is also employed to refine DOA estimations and reduce quantization errors. Simulation results demonstrate that the proposed approach achieves significantly higher estimation accuracy and resolution than conventional methods and current DL models under varying SNR and snapshot conditions. In addition, it offers distinct advantages in terms of generalization and computational efficiency.

## 1. Introduction

Direction-of-Arrival (DOA) estimation constitutes a central focus in array signal processing and is widely applied in fields including radar detection, wireless communication, electronic countermeasures, acoustic direction finding, and astronomy. The primary objective of DOA estimation is to accurately determine the angles of arrival of incoming signals received by an antenna array, thereby providing critical parameters for further processing tasks such as target tracking and localization [1,2,3]. DOA estimation is a fundamental technology underpinning a wide range of applications, including modern wireless communication systems, intelligent transportation, Unmanned Aerial Vehicle (UAV) coordination, and future 6G networks [4,5,6]. For instance, in multiple-input multiple-output (MIMO) communication systems, accurate DOA estimation can significantly enhance beamforming performance and improve spatial multiplexing capabilities [7,8,9]. Similarly, in radar systems, it supports multi-target detection and tracking, thereby improving the accuracy and robustness of target identification. Accordingly, achieving high-precision and low-complexity DOA estimation under complex conditions has remained a critical research focus in both academia and industry [10,11].

Over the past decades, DOA estimation has been extensively studied, leading to the development of various classical methods. One of the earliest algorithms is the conventional beamforming (CBF) method, whose core principle is to exploit the array antenna’s response to signals from different directions and estimate the angles of arrival by constructing a spatial beam pattern [12,13]. However, the performance of this method is constrained by the Rayleigh resolution limit, making it difficult to distinguish signal sources with small angular separations. Additionally, it is highly susceptible to noise in low signal-to-noise ratio (SNR) environments, resulting in a significant degradation of estimation accuracy [14,15,16]. In response, subspace decomposition-based super-resolution algorithms were introduced in the 1980s to address these shortcomings [17,18]. The Multiple Signal Classification (MUSIC) algorithm exploits the orthogonality between the signal subspace and the noise subspace. It performs eigenvalue decomposition of the covariance matrix to extract the signal subspace and estimates the DOA by searching for peaks in the spatial spectrum [19,20]. Nevertheless, MUSIC suffers from high computational complexity in large-scale arrays and considerable performance degradation under coherent source conditions, limiting its practical applicability [21]. To address these challenges, the Estimation of Signal Parameters via Rotational Invariance Techniques (ESPRIT) algorithm was introduced in 1985. This method exploits the rotational invariance between subarrays and directly estimates the DOA parameters using a parameter matrix derived from eigenvalue decomposition, thus eliminating the need for spectral peak search and improving computational efficiency [22,23]. Other DOA estimation algorithms have also been developed in the statistical signal processing area, including the Minimum Variance Distortionless Response (MVDR) method. Although this method can enhance the accuracy of DOA estimation, it is sensitive to noise and the number of snapshots [24,25,26]. Similarly, the Maximum Likelihood (ML) method provides theoretically optimal estimation performance but suffers from prohibitively high computational complexity, making it unsuitable for real-time applications [27,28,29].

Despite the satisfactory performance of traditional DOA estimation methods under certain conditions, they exhibit notable limitations. These include poor estimation accuracy in low SNR environments, limited snapshots, coherent signal scenarios, and complex propagation channels. Moreover, they suffer from excessive computational complexity and strong sensitivity to parameter selection, which significantly hinders their practical deployment [30,31]. In recent years, with the rapid development of artificial intelligence technologies, deep learning (DL) has achieved remarkable success in fields such as computer vision, natural language processing, and medical image analysis. It has also been gradually introduced into DOA estimation research to enhance model adaptability under complex environments [32,33,34]. Unlike conventional model-based approaches, DL adopts a data-driven, end-to-end framework to directly learn the input–output relationship, offering improved generalization and robustness [9,35]. DL-based DOA estimation methods are typically categorized into classification and regression types. The classification methods discretize the angular space into several intervals and model the estimation as a classification problem, where neural networks are trained to predict the interval corresponding to the incident signal angle [36,37]. For example, DOA estimation algorithms based on convolutional neural networks (CNNs) utilize their spatial feature extraction capabilities and apply fully connected layers for classification, thereby improving resolution [36,38]. In contrast, regression-based methods predict the continuous DOA angles directly through deep neural networks, thereby circumventing the accuracy loss due to discretization. However, this also increases the complexity of signal feature extraction and processing [39,40]. Although DL has shown initial progress in the field of DOA estimation, various challenges still need to be addressed. Firstly, existing DL-based DOA estimation methods show limited feature extraction ability and stability when confronted with complex signal source scenarios, frequently encountering challenges such as gradient vanishing and feature degradation. Second, commonly used loss functions—primarily mean squared error (MSE) and binary cross-entropy (BCE)—fail to account for spatial spectrum sparsity and treat all samples equally, regardless of their confidence levels. This can lead to overfitting on easily learned samples and poor detection of low-confidence or weak signal sources. Additionally, many DL methods exhibit instability under extreme conditions like low SNR, small snapshot numbers, or high adjacent source density, generally lacking generalization ability, robustness, and computational efficiency, thereby constraining their practical application.

In this paper, a DL-based DOA estimation method is proposed. The approach employs a deep convolutional neural network enhanced by a cross-residual structure, coupled with a Long Short-Term Memory (LSTM) network to effectively capture both spatial and temporal features. An end-to-end training framework is employed, integrating a composite FD loss function that combines Focal Loss and Dice Loss to improve the sensitivity of model to low-confidence samples and promote sparsity in spatial spectrum predictions. Furthermore, peak detection and quadratic interpolation-based angle regression are introduced to mitigate quantization errors and further refine estimation accuracy. Simulation results confirm that the proposed method consistently outperforms traditional and existing DL approaches across various SNR levels, snapshot numbers, and resolution, offering a robust and high-precision solution for DOA estimation. The main contributions of this paper are summarized as follows:

- A CRDCNN-LSTM architecture is proposed, designed for joint spatial–temporal feature fusion. The convolutional module includes multi-level cross-residual connections that mitigate the issues of traditional single-path feature flow, enhancing both multi-scale feature representation and feature diversity. The six-layer stacked CRDCNN configuration allows each layer to retain its features while passing the outputs of previous layers to subsequent ones, ensuring effective deep information propagation and addressing challenges such as gradient vanishing. The LSTM module captures temporal dependencies, which significantly enhances the robustness of DOA estimation under noisy conditions.
- An FD loss function is designed, integrating Focal Loss and Dice Loss. Focal Loss introduces a modulation factor to down-weight easy samples and emphasize hard samples, thereby reducing overfitting and improving the detection of low-confidence or weak signals. The Dice Loss component optimizes the sparsity and distribution consistency of the spatial spectrum by quantifying the overlap between predicted and true spectra. The weighted combination of these two losses accelerates model convergence and improves generalization, particularly under low SNR or data imbalance conditions, thus enhancing the accuracy and robustness of DOA estimation.
- To mitigate the impact of angle discretization and associated quantization errors in the estimation results, this paper introduces a post-processing strategy that combines peak detection with quadratic interpolation. The method first detects peaks in the spatial spectrum output by the network to locate prominent responses corresponding to signal directions. Then, quadratic interpolation is used to refine these peak positions to sub-pixel accuracy, enabling high-precision estimation in the continuous angular domain. This approach effectively alleviates quantization-induced localization errors and significantly enhances the resolution and stability of DOA estimation, particularly in scenarios involving closely spaced sources.

The remainder of this paper is organized as follows. Section 2 introduces the signal model based on the uniform linear array (ULA) and discusses the signal information contained in the spatial spectrum. Section 3 gives the architecture of the proposed model in detail. The advantages and disadvantages of the proposed framework are explored and compared with other common methods using simulated experiments in Section 4. Finally, Section 5 concludes the paper.

## 2. Singal Model

**Figure 1:**
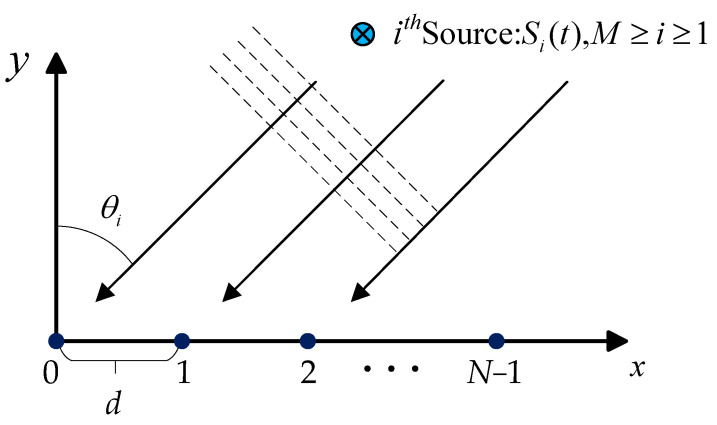
Uniform linear array structure.

Consider a ULA consisting of *N* elements with an inter-element spacing of *d*. In the spatial domain, *M* uncorrelated far-field narrowband signals impinge upon the array from various directions, as illustrated in Figure 1.

Denote the number of snapshots per element as *T*. The signal received by the *n*-th array element at time instant *t* can be expressed as

(1) $$x_{n}(t)=\sum_{i=1}^{M}s_{i}(t)e^{-j\frac{2\pi }{\lambda }(n-1)d\sin\theta_{i}}+n_{n}(t),\;n=1,2,\ldots ,N$$

where λ is the signal wavelength, defined by λ=c/f, where *c* represents the speed of light and *f* is the frequency of the signal. $s_{i}(t)$ denotes the envelope of the *i*-th narrowband signal at time *t*, containing both amplitude and phase information. $\theta_{i}$ indicates the direction of arrival of the *i*-th signal, while $n_{n}(t)$ represents additive noise, typically assumed to be zero-mean complex Gaussian white noise with known variance. To facilitate matrix-based derivation, let

(2) $$x(t)={\left[\begin{array}{c} x_{1}(t),x_{2}(t),\ldots ,x_{N}(t) \end{array}\right]}^{T}\in {\mathbb{C}}^{N\times 1}$$

where ${\left(\cdot \right)}^{T}$ denotes the transpose operation. Accordingly, the above expression can be rewritten as

(3) $$x(t)=\sum_{i=1}^{M}s_{i}(t)a(\theta_{i})+n(t)$$

where n(t) is the noise vector, and $a(\theta_{i})$ denotes the array manifold vector of the *i*-th signal arriving from angle $\theta_{i}$, which is given by

(4) $$a(\theta_{i})={\left[\begin{array}{c} 1,e^{-j\frac{2\pi }{\lambda }d\sin\theta_{i}},e^{-j\frac{4\pi }{\lambda }d\sin\theta_{i}},\ldots ,e^{-j\frac{2\pi }{\lambda }(N-1)d\sin\theta_{i}} \end{array}\right]}^{T}$$

by concatenating the array manifold vectors of all *M* signals, we obtain the following:

(5) $$A(\theta )=\left[\begin{array}{c} a(\theta_{1}),a(\theta_{2}),\ldots ,a(\theta_{M}) \end{array}\right]\in {\mathbb{C}}^{N\times M}$$

All signal envelopes at time *t* can be arranged into the following vector:

(6) $$s(t)={\left[\begin{array}{c} s_{1}(t),s_{2}(t),\ldots ,s_{M}(t) \end{array}\right]}^{T}$$

thus, Equation (1) can be further expressed as follows:

(7) x(t)=A(θ)s(t)+n(t)

In the case of offline or batch processing, *T* snapshots can be collected at times t=1,2,…,T. In practice, the array covariance matrix $R=E[x(t)x{\left(t\right)}^{H}]$ is replaced by the sample covariance matrix $\hat{R}$, which is computed from the *T* snapshots of the array output x(t) and is given by

(8) $$\hat{R}=\frac{1}{T}\sum_{t=1}^{T}x(t)x{\left(t\right)}^{H}$$

where ${\left(\cdot \right)}^{H}$ indicates the conjugate transpose operation. The covariance matrix $\hat{R}$ reflects the statistical characteristics of the signals and noise across the array elements and provides essential information for subsequent spatial spectrum calculation. The steering vector based on $\hat{R}$ is then defined as

(9) $$a(\theta )=\exp\left(-j\frac{2\pi }{\lambda }d\sin(\theta )\right)$$

where $d={\left[\begin{array}{c} 0,d,2d,\ldots ,(N-1)d \end{array}\right]}^{T}$ represents the spatial positions of the elements in the ULA. If we define

(10) $$W(\theta )=a(\theta )a^{H}(\theta )$$

the matrix W(θ) and the sample covariance matrix $\hat{R}$ can be vectorized using vec(·), resulting in the column vectors vec(W(θ)) and $\mathrm{vec}(\hat{R})$. Subsequently, the beam output amplitude at scanning angle is calculated as follows:

(11) $$Z(\theta )=\left|\mathrm{vec}{\left(W(\theta )\right)}^{H}\mathrm{vec}(\hat{R})\right|$$

by iteratively scanning θ across the predefined angular grid $\Theta =\{\theta_{1},\theta_{2},\ldots ,\theta_{L}\}$, the full spatial spectrum distribution $\left\{Z(\theta_{1}),Z(\theta_{2}),\ldots ,Z(\theta_{L})\right\}$ is derived. The spatial spectrum generally shows significant peaks near the true incident angles of the signals, which can be used as feature inputs for neural network training.

In the context of data-driven DOA estimation, the actual signal directions are labeled during network training or testing by assigning 0-1 label vectors to the samples. Specifically, if the discrete angular grid Θ comprises *L* discrete angles $\left\{\theta_{1},\theta_{2},\ldots ,\theta_{L}\right\}$, a label vector of length *L* is constructed as follows:

(12) $$Y={\left[\begin{array}{c} y_{1},y_{2},\ldots ,y_{L} \end{array}\right]}^{T}$$

where

(13) $$y_{l}=\left\{\begin{array}{l} 1,\;\theta_{l}\;\mathrm{is}\;\mathrm{the}\;\mathrm{angle}\;\mathrm{of}\;\sin\mathrm{gal}\\ 0,\;\mathrm{else} \end{array}\right.$$

For multi-source situations, one-hot encoding is applied by assigning 1 to the positions corresponding to each true signal direction in the label vector. The resulting 0–1 labels Y serve to guide the network in distinguishing signal components from noise during training, enhancing its ability to accurately localize source peaks in the spatial spectrum when handling new signals.

## 3. Proposed Method

This paper presents a DL-based DOA estimation approach, with its core being the construction of the Cross-Residual Deep Convolutional and Long Short-Term Memory Network. The network employs a CRDCNN to extract spatial features from the input signals and integrates an LSTM module for temporal sequence modeling, thereby improving DOA estimation performance under low SNR conditions, limited snapshot availability, and complex signal environments. According to Equation (11), the sample features are described by the spatial spectrum, and the feature matrix input to the network is defined as follows:

(14) $$X=\left\{Z\left(\theta_{1}\right),Z\left(\theta_{2}\right),\ldots ,Z\left(\theta_{L}\right)\right\}\in {\mathbb{R}}^{B\times 1\times L}$$

**Figure 2:**
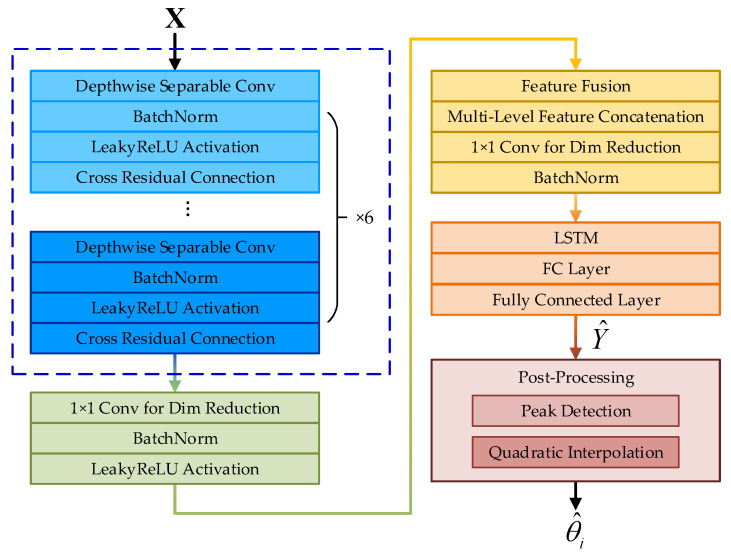
Proposed DOA estimation method.

In this formulation, *B* denotes the batch size and *L* represents the number of discrete angular grid points. The proposed method operates as follows: The input features are first fed into the CRDCNN to perform multi-scale feature extraction. The LSTM module then captures the temporal dependencies of the sequential features, enabling the model to learn the evolution of DOA signals across different numbers of snapshots and enhancing temporal stability. Finally, a fully connected layer is used to generate the output, followed by a post-processing step that extracts the peak positions to obtain the final DOA estimates. The overall framework is illustrated in Figure 2, where the blue dashed box denotes the CRDCNN.

### 3.1. Network Architecture

#### 3.1.1. Cross-Residual Deep Convolutional Network

For DOA estimation, the input features X encompass abundant spatial information, necessitating efficient extraction of the discriminative characteristics between signals and noise. Conventional DCNNs tend to suffer from problems such as information loss and gradient vanishing as network depth increases. Therefore, a cross-residual structure is introduced to enhance feature extraction and improve inter-layer information propagation. The CRDCNN consists of six cross-residual deep convolutional layers, each of which employs depthwise separable convolution to reduce computational complexity while maintaining effective feature representation capability. Let the input feature of the *l*-th layer be $X_{l}$, the output as $H_{l}$, and the convolution process is formulated as

(15) $$H_{l}=\sigma_{L}\left(\mathrm{BN}\left({\mathrm{Conv}}_{\mathrm{depthwise}}(X_{l})+{\mathrm{Conv}}_{\mathrm{pointwise}}(X_{l})\right)\right)$$

where ${\mathrm{Conv}}_{\mathrm{depthwise}}$ denotes depthwise convolution, ${\mathrm{Conv}}_{\mathrm{pointwise}}$ denotes pointwise convolution, BN represents batch normalization, and $\sigma_{L}$ is the LeakyReLU activation function. After feature extraction at each layer, CRDCNN fuses information ${\tilde{H}}_{l}$ through cross-residual connections, which can be written as

(16) $${\tilde{H}}_{l}=H_{l}+\sum_{i=1}^{l-1}\alpha_{i}H_{i}$$

where $\alpha_{i}$ is a learnable parameter that controls the residual information flow between different layers.

**Figure 3:**
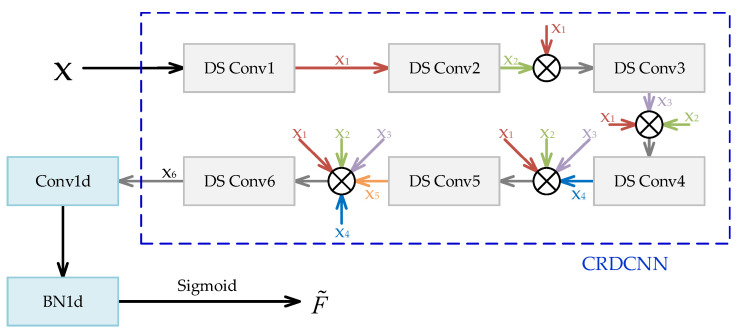
Proposed CRDCNN architecture.

The CRDCNN consists of six layers designed for progressive feature extraction, gradually enhancing the network’s spatial perception ability. The specific architecture is depicted in Figure 3. The first layer employs depthwise separable convolutions with an increased number of channels and large receptive fields to capture global information. As the network deepens, both the dilation rate and kernel size are progressively reduced across layers, facilitating a transition from coarse to fine-grained feature extraction. Each layer receives inputs not only from the immediately preceding layer but also integrates features from all preceding layers, ensuring the full utilization of multi-scale representations. In the sixth layer, the number of channels is further expanded, and small-scale convolutions are applied to strengthen the expressiveness of the final features based on the fused multi-layer information.

During the final fusion stage of CRDCNN, the outputs from all layers are concatenated and subjected to a 1 × 1 convolution for dimensionality reduction, resulting in the final feature representation:

(17) $$\tilde{F}=\sigma_{L}\left(\mathrm{BN}\left({\mathrm{Conv}}_{1\times 1}\left(\left[H_{1},H_{2},\ldots ,H_{6}\right]\right)\right)\right)$$

where $\left[H_{1},H_{2},\ldots ,H_{6}\right]$ denotes the concatenation of outputs from all layers. The cross-residual structure enhances inter-layer information flow, promoting feature reuse and improving the model’s representational capacity. The use of depthwise separable convolutions reduces computational complexity, making the model more trainable on large-scale datasets. Additionally, the combination of batch normalization (BN) and LeakyReLU effectively mitigates the gradient vanishing problem, improving training stability.

#### 3.1.2. Long Short-Term Memory Network and Output Layer

**Figure 4:**
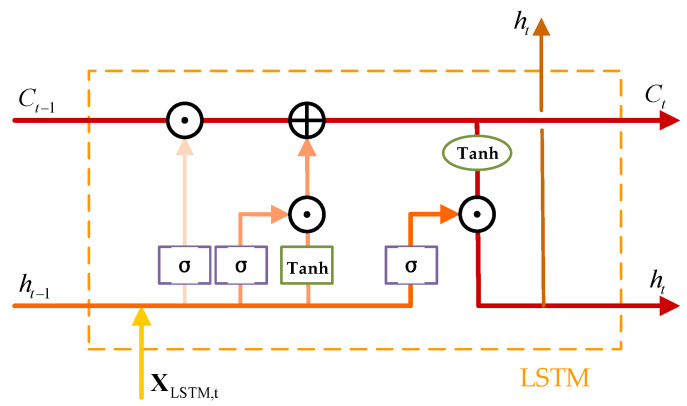
Computation diagram of LSTM unit.

Due to the temporal correlation of DOA signals across snapshots, temporal sequence modeling is essential to improve estimation stability. To this end, an LSTM network is employed to enhance the features extracted by the CRDCNN. LSTM is an improved variant of the recurrent neural network (RNN), featuring a memory cell (cell state) that retains historical information, along with input, forget, and output gates that regulate the flow and updating of information. The structure is illustrated in Figure 4. In this figure, ⊕ is used to indicate element-wise addition, while ⊙  represents element-wise multiplication.

The output dimension of $\tilde{F}$ is set to ${\mathbb{R}}^{B\times 16\times L}$, and the corresponding input feature matrix for the LSTM is given by

(18) $$X_{\mathrm{LSTM}}={\tilde{F}}^{T}\in {\mathbb{R}}^{B\times 16\times L}$$

where 16 is the number of feature channels. The LSTM computation leverages the temporal evolution patterns of DOA signals. Given that the snapshot number *T* influences the statistical properties of the signals in DOA estimation, the LSTM utilizes feature inputs across time steps to capture the temporal sequence correlations. Let the LSTM hidden state dimension be $h_{t}$ and the cell state be $c_{t}$, the operation of LSTM is described in Algorithm 1.
**Algorithm 1:** LSTM Operations at Each Time Step**Inputs:** $X_{\mathrm{LSTM},t}$, $h_{t-1}$, $c_{t-1}$, $W_{f},W_{i},W_{c},W_{o}$, $b_{f},b_{i},b_{c},b_{o}$ **Output:** $h_{t}$, $c_{t}$Forget Gate:      $f_{t}=\sigma_{S}(W_{f}[h_{t-1},X_{\mathrm{LSTM},t}]+b_{f})$Input Gate:     $i_{t}=\sigma_{S}(W_{i}[h_{t-1},X_{\mathrm{LSTM},t}]+b_{i})$      ${\tilde{c}}_{t}=\tanh(W_{c}[h_{t-1},X_{\mathrm{LSTM},t}]+b_{c})$Update Cell State:      $c_{t}=f_{t}⊙c_{t-1}+i_{t}⊙{\tilde{c}}_{t}$Output Gate:      $o_{t}=\sigma_{S}(W_{o}[h_{t-1},X_{\mathrm{LSTM},t}]+b_{o})$Compute Hidden State:      $h_{t}=o_{t}⊙\tan h(c_{t})$**Return** $h_{t}$,$c_{t}$

Where $W_{f},W_{i},W_{c},W_{o}$ and $b_{f},b_{i},b_{c},b_{o}$ are the learnable parameters of the LSTM, $\sigma_{S}$ denotes the Sigmoid activation function and tanh represents the hyperbolic tangent function. In this paper, a bidirectional LSTM (BiLSTM) structure is adopted to fully exploit temporal information. The computation of the BiLSTM is formulated as

(19) $$h_{t}^{\to }={\mathrm{LSTM}}_{\mathrm{forward}}(X_{\mathrm{LSTM},t},h_{t-1})$$

(20) $$h_{t}^{\leftarrow }={\mathrm{LSTM}}_{\mathrm{backward}}(X_{\mathrm{LSTM},t},h_{t+1})$$

(21) $$H_{t}=[h_{t}^{\to },h_{t}^{\leftarrow }]$$

By capturing global temporal dependencies from both past and future time steps, the bidirectional LSTM improves the robustness of DOA estimation. A fully connected (FC) layer is subsequently applied after the LSTM to yield the final angle estimation:

(22) $$\hat{Y}=\sigma_{S}(W_{\mathrm{out}}H_{t}+b_{\mathrm{out}})$$

where $W_{\mathrm{out}}$ and $b_{\mathrm{out}}$ are the learnable parameters of the output layer, and $\hat{Y}$ denotes the final output pseudo-spatial spectrum.

### 3.2. Loss Function

The design of the loss function is critical in training DL-based DOA estimation models. Conventional loss functions, such as mean squared error (MSE) or binary cross-entropy (BCE), may not adequately reflect the specific challenges of DOA estimation, particularly in environments with low SNR, few snapshots, or coherent signal sources. Under such conditions, networks are prone to noise interference, leading to reduced estimation accuracy. To mitigate this, the proposed method employs a combination of Focal Loss and Dice Loss, termed FD Loss, to increase attention to low-confidence samples, enforce sparsity in the predicted spatial spectrum, and enhance the robustness of the DOA estimation.

Focal Loss is an advanced loss function based on cross-entropy, developed to handle class imbalance in tasks such as object detection. In DOA estimation, true DOA positions occupy only a small fraction of the spatial spectrum, while the majority of positions correspond to near-zero probabilities. This highly imbalanced distribution causes standard cross-entropy to overemphasize high-probability regions and neglect low-probability areas. Focal Loss mitigates this issue by introducing a modulation factor that decreases the influence of well-classified samples and increases the focus on difficult, low-confidence samples, thereby improving DOA estimation accuracy. The mathematical formulation is

(23) $$L_{\mathrm{FL}}=-\alpha {\left(1-p_{t}\right)}^{\gamma }\log(p_{t})$$

where $p_{t}$ denotes the predicted probability of the target class by the network, defined as follows:

(24) $$p_{t}=y_{i}p+(1-y_{i})(1-p)$$

where $y_{i}\in Y$ denotes the ground-truth DOA label, while *p* represents the predicted probability at angle $\theta_{i}$. For positive samples (locations corresponding to DOA), $p_{t}=p$; for negative samples (non-DOA locations), $p_{t}=1-p$. The modulation factor ${\left(1-p_{t}\right)}^{\gamma }$ in Focal Loss controls the loss weighting for samples with varying confidence levels. When $p_{t}$ approaches 1 (indicating accurate prediction), the loss contribution diminishes, reducing the influence of easy samples. Conversely, for low-confidence samples (small $p_{t}$), the loss increases, prompting the model to focus more on these difficult cases and enhancing DOA estimation stability. The parameter γ adjusts the modulation strength, larger values accentuate hard samples, while smaller values make Focal Loss converge to cross-entropy loss.

The spatial spectrum in DOA estimation is characterized by sparsity, where true DOA targets occupy only a small fraction, and the majority corresponds to background regions (non-DOA positions), resulting in significant class imbalance. The incorporation of Focal Loss allows the model to dynamically adjust the weighting of different samples, ensuring greater focus on DOA signal positions and improving detection performance, particularly in low SNR environments.

Dice Loss, initially introduced in the context of medical image segmentation, is designed to address extreme class imbalance. The central idea is to calculate the Dice similarity coefficient between the predictions of model and the true DOA labels, thereby quantifying their alignment and optimizing estimation performance. Its mathematical expression is formulated as

(25) $$L_{\mathrm{Dice}}=1-\frac{2\sum p_{i}y_{i}+\epsilon }{\sum p_{i}+\sum y_{i}+\epsilon }$$

where $p_{i}$ refers to the predicted probability at the *i*-th angular position, and ϵ is a small constant (e.g., $10^{-6}$) introduced to avoid division by zero. Dice Loss enhances DOA estimation accuracy by maximizing the similarity between predicted outputs and true DOA labels via the Dice coefficient. Given the sparse nature of DOA labels, Dice Loss effectively aligns the predicted spatial spectrum with the ground-truth DOA distribution and mitigates the impact of background noise. Moreover, Dice Loss offers stable gradients, enabling faster convergence during early training phases and facilitating efficient learning of DOA features even when data are limited.

In the tasks of DOA estimation, applying either Focal Loss or Dice Loss independently may not effectively balance learning focus on low-confidence samples and global optimization of the spatial spectrum. Therefore, FD Loss is introduced by weighted fusion of Focal Loss and Dice Loss to enhance estimation precision. The formulation is as follows:

(26) $$L_{\mathrm{FD}}=\alpha L_{\mathrm{FL}}+\beta L_{\mathrm{Dice}}$$

where α and β correspond to the weights assigned to Focal Loss and Dice Loss, respectively, balancing their influence throughout training. According to Equation (22), the network output pseudo-spatial spectrum is denoted by $\hat{Y}$, and the true DOA labels obtained from Equation (12) are denoted as Y. Thus, the FD loss function is expressed as

(27) $$L_{\mathrm{FD}}=\alpha L_{\mathrm{FL}}(\hat{Y},Y)+\beta L_{\mathrm{Dice}}(\hat{Y},Y)$$

The first term indicates the Focal Loss, which applies higher weights to low-confidence samples at DOA positions, enhancing the ability of network to capture difficult cases. The second term refers to the Dice Loss, which optimizes the overall correspondence between the predicted and true DOA spatial spectra, thereby improving prediction accuracy.

### 3.3. Post-Processing

After the input signals are processed by the CRDCNN-LSTM network, a continuous probability distribution $\hat{Y}$ is produced. Using $\hat{Y}$ directly for DOA estimation may lead to significant inaccuracies. To address this, a post-processing step is adopted to extract the final estimated DOA angles. As the network output consists of unnormalized activations, it is necessary to apply a Sigmoid function to convert them into probability values:

(28) $$\Psi =\sigma (\hat{Y})$$

This operation maps the raw prediction values into the [0, 1] interval, giving the network output probabilistic significance and facilitating subsequent DOA angle estimation. In this work, two steps—peak detection and angle refinement—are applied to extract peak values from the pseudo-spatial spectrum Ψ and determine the angles of the DOA signals. First, peak detection is used to identify local maxima within Ψ:

(29) $$\Theta_{\mathrm{peak}}=\{\theta_{i}|\Psi_{i}>\Psi_{i-1}\;\&\;\Psi_{i}>\Psi_{i+1},i\in \{2,\ldots ,L-1\}\}$$

Specifically, when the probability value $\Psi_{i}$ at angle $\theta_{i}$ is greater than those at its neighboring grid points, $\theta_{i}$ is identified as a peak DOA angle. Through peak detection, an initial set of candidate DOA angles can be obtained. However, since the discrete angular grid Θ is finite, the true DOA angles may not exactly coincide with the grid points. To address this, an angle refinement process is applied to the detected peaks to further improve estimation accuracy. The core idea of angle refinement is to perform quadratic interpolation around the detected peak positions to estimate the actual DOA angles. Let the network outputs at a peak position $\theta_{i}$ and its two adjacent points be $\Psi_{i-1},\Psi_{i},\Psi_{i+1}$, respectively. The final estimated DOA angle $\hat{\theta }$ at the peak position is obtained using quadratic interpolation as follows:

(30) $$\hat{\theta }=\theta_{i}+\frac{\Psi_{i+1}-\Psi_{i-1}}{2(\Psi_{i-1}-2\Psi_{i}+\Psi_{i+1})}\cdot \bar{\theta }$$

where $\bar{\theta }$ denotes the grid step size, i.e., the interval between two adjacent angular grid points. The derivation of this formula is based on fitting the probability distribution near the peak with a quadratic function and solving for its extremum to obtain a refined DOA estimate. This angle regression technique effectively eliminates the quantization errors caused by grid discretization and leads to a significant improvement in the resolution of the final DOA estimation.

## 4. Simulation Results

### 4.1. Data Generation

In this section, signal samples are generated using a uniform linear array, as illustrated in Figure 1. The array configuration includes *M* = 8 elements with an element spacing of d=λ/2, and the number of sources is *N* = 2. The number of snapshots is *T* = 256. The angle grid for the simulated array covers the range Θ∈ [−75°,75°], partitioned at intervals of 1°. Twenty distinct angular separations are considered, covering intervals of 2°,4°,…,40°. Signal samples are generated corresponding to each angular separation $\Delta \theta_{q}$, and the angles of the generated samples span

(31) $$\begin{array}{c} \theta_{q}^{\prime }=-75{}^{\circ}+l_{q}\cdot 1{}^{\circ}\\ \theta_{q}^{\prime \prime }=-75{}^{\circ}+l_{q}\cdot 1{}^{\circ}+\Delta \theta_{q} \end{array}$$

The index $l_{q}$ ranges from $\;0,\ldots ,D_{q}$, where $D_{q}=150-\Delta \theta_{q}+1$. A total of 2600 angle sample sets are generated for the 20 angular intervals. The signal-to-noise ratios are randomly chosen within the range [−10 dB, 10dB], and the corresponding labels are derived from the 0–1 spatial spectrum obtained via Equation (12). Overall, 26,000 simulation samples are generated, covering various angular separations and noise levels.

### 4.2. DOA Estimation Performance

**Figure 5:**
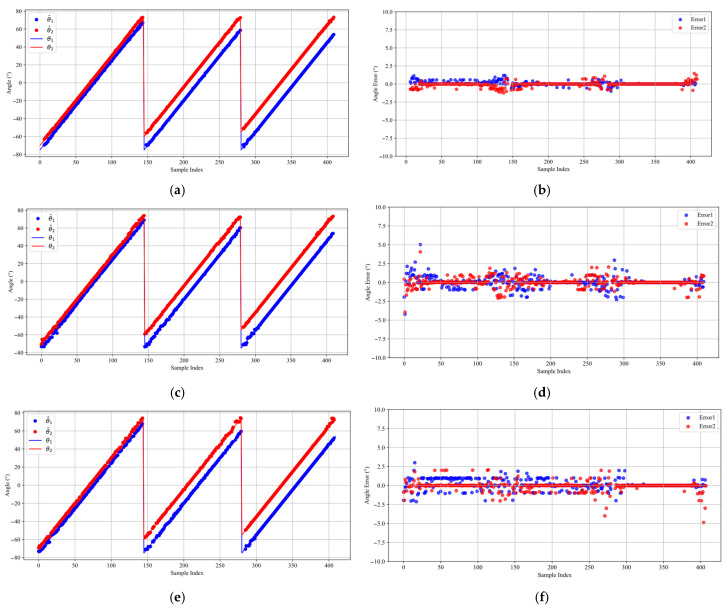
DOA estimation performance of different DL methods. (**a**) Proposed method for DOA estimation. (**b**) Estimation errors of Proposed. (**c**) DCNN for DOA estimation. (**d**) Estimation errors of DCNN. (**e**) Res-DCNN for DOA estimation. (**f**) Estimation errors of Res-DCNN.

Two far-field narrowband independent signals with identical SNRs of 0 dB are considered, with angular intervals of {5°,15°,25°}. For each angular separation, Equation (31) is employed to generate the corresponding angle values, which are used to produce the test samples. Following the reconstruction of the spatial spectrum, the DOA estimates for each test sample are calculated according to Equation (30). The DOA estimation results obtained by the three DL methods are displayed in Figure 5, with the estimated DOAs shown on the left and the corresponding estimation errors on the right.

Figure 5a,b illustrate that the proposed method demonstrates exceptional performance in DOA estimation tasks under typical scenarios. It achieves consistently high estimation accuracy across all angular separations and significantly surpasses competing methods, especially in the presence of closely spaced signal sources. Furthermore, the estimation errors are consistently maintained within ±1° even in proximity to the grid boundaries. This superior performance is primarily due to the FD loss function optimization, which allows the model to leverage boundary samples more effectively during training and improves its generalization across the full range of angles. In comparison, while the DCNN method (Figure 5c,d) exhibits generally balanced estimations, its accuracy is notably lower than that of the proposed method, with errors exceeding 2.5° close to the grid boundaries. Despite the enhanced feature extraction capability of the Res-DCNN method (Figure 5e,f), the error distribution is relatively irregular, particularly at the 5° angular separation.

### 4.3. Generalization Capability Under Multi-Source Scenarios

In order to assess the generalization performance of various DOA estimation algorithms under multi-source conditions, this experiment generates sample data in scenarios with multiple signal sources. The proposed approach is compared with other DL methods as well as traditional techniques such as L1-SVD, MUSIC, and MVDR. Specifically, the number of sources is set to *M* = 5, with SNR = 0 dB, *T* = 100, and source angles positioned at [−46°,−23°,0°,23°,46°]. The DOA estimation results are visualized by plotting the spatial spectrum in a 3D space.

**Figure 6:**
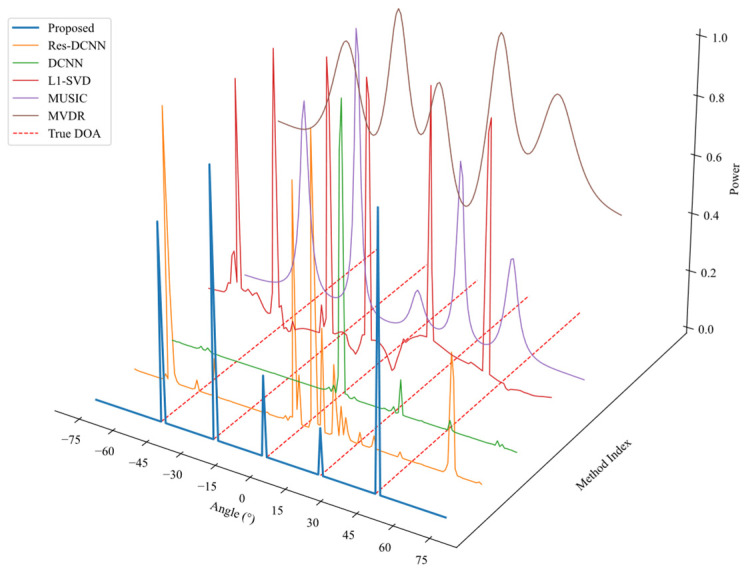
Power spectrum comparison of different DOA estimation methods.

As shown in Figure 6, significant differences are observed in the spatial spectrum estimation results of various DOA estimation algorithms under a multi-source environment. Traditional methods such as L1-SVD produce sharp spectral peaks but suffer from the presence of spurious peaks. Both MUSIC and MVDR generate relatively clear spectral peaks, with MUSIC exhibiting sharper peaks; however, these methods require prior knowledge of the number of signal sources. In contrast, the proposed method produces sharper spectral peaks than MUSIC without needing the number of sources as a prior. The DCNN model yields only a three dominant peak, indicating limited generalization capability. Although the Res-DCNN model enhances feature extraction, it still suffers from missed sources and spurious peaks.

**Figure 7:**
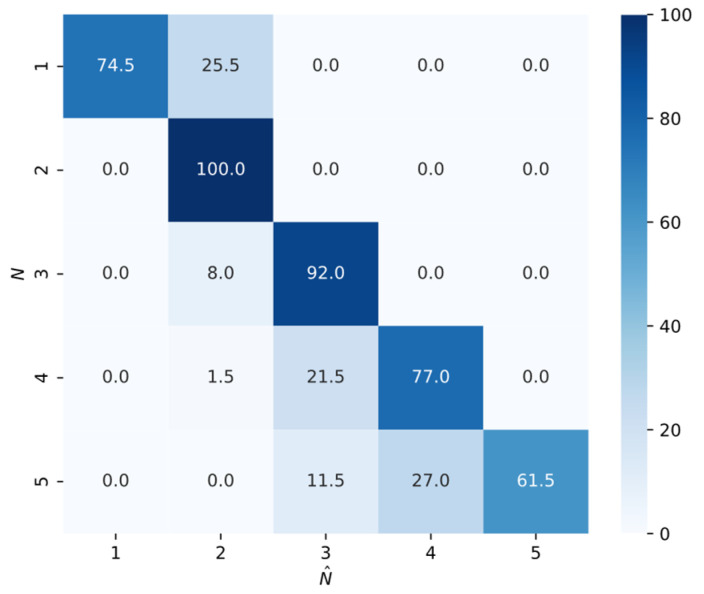
Confusion matrix under unknown source number conditions.

To further verify its generalization, the proposed model was tested on data with unknown source numbers $M\in \left\{1,2,3,4,5\right\}$, with 200 samples generated for each case and no prior knowledge of *M* provided. As shown in Figure 7, the confusion matrix illustrates that most predictions align with the ground truth, especially for *M* = 2 to 4. Even for more complex cases with *M* = 1 and 5, the model remains accurate despite being trained only on two-source samples, demonstrating strong robustness and generalization beyond its training distribution.

The method incorporates cross-residual convolutional layers and LSTM networks to exploit both spatial and temporal information, leading to enhanced DOA feature learning and effective suppression of false peaks. The integration of the FD loss function further optimizes key angle detection and noise robustness, ensuring the method achieves superior spectral peak definition, high resolution, and strong generalization performance.

### 4.4. Statistical Performance Analysis

In the DOA estimation experiments, we use the root mean square error (RMSE) as the key evaluation metric to assess the estimation accuracy of different models under different experimental setups [41]. The formula for RMSE is as follows:

(32) $$\mathrm{RMSE}=\sqrt{\frac{1}{K\cdot M}\sum_{k=1}^{K}\sum_{m=1}^{M}{\left({\hat{\theta }}_{m,k}-\theta_{m}\right)}^{2}}$$

where *K* specifies the number of Monte Carlo runs, *M* is the number of signal sources, ${\hat{\theta }}_{m,k}$ denotes the estimated direction of arrival for the *m*-th signal in the *k*-th experiment, and $\theta_{m}$ corresponds to the true angle. In order to incorporate a more comprehensive lower bound evaluation of the proposed method, we have additionally included the Cramér–Rao Lower Bound (CRLB) as a baseline alongside the previously discussed comparison methods [42].

#### 4.4.1. Impact of Signal-to-Noise Ratio on Estimation Accuracy

In this experiment, the number of snapshots is set to *T* = 256 and signal source angles of $\theta_{1}=11{}^{\circ}$ and $\theta_{2}=23{}^{\circ}$. The SNR is selected as the experimental variable, varying from −10 dB to 10 dB in steps of 2 dB, resulting in 11 SNR scenarios. For each scenario, 600 Monte Carlo experiments are conducted to ensure statistical reliability. Under these conditions, only the noise level is altered to assess its impact on DOA estimation accuracy.

**Figure 8:**
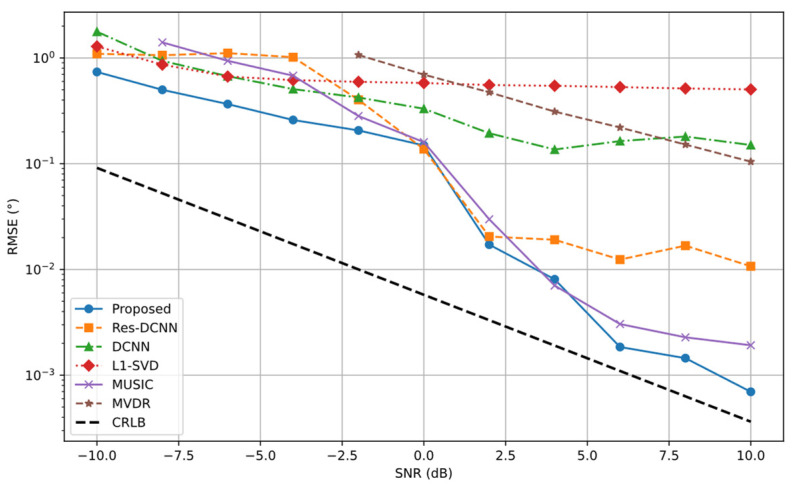
RMSE curves of different SNR for various methods.

Figure 8 shows that RMSE decreases as SNR increases for most methods. The proposed method maintains the lowest RMSE across the entire SNR range and gradually approaches the CRLB when SNR exceeds 0 dB. Under low SNR conditions (SNR < 0 dB), it achieves significantly lower errors and a more rapid RMSE reduction, indicating strong robustness to noise. This advantage results from the cooperative extraction of spatial and temporal features by the CRDCNN-LSTM framework, along with the effective role of the FD loss function in key angle detection and noise reduction. By contrast, although the RMSE of the Res-DCNN and DCNN methods decreases with increasing SNR, both suffer from large estimation errors under low SNR conditions (notably SNR < −5 dB), reflecting limited robustness. The traditional L1-SVD and MUSIC algorithms similarly perform inadequately at low SNRs, with L1-SVD exhibiting consistently high RMSE and MUSIC being highly susceptible to noise, offering marginal advantages only at high SNRs. The MVDR method shows high RMSE under all SNR conditions, particularly struggling to deliver effective DOA estimation in low SNR environments.

#### 4.4.2. Impact of Snapshot Number on Estimation Accuracy

In this experiment, the SNR is set to 0 dB, and the source angles are configured as $\theta_{1}=11{}^{\circ}$ and $\theta_{2}=23{}^{\circ}$. The experimental variable is the number of snapshots *T*, varied from 50 to 500 with a step of 50, resulting in multiple snapshot conditions. A total of 600 Monte Carlo trials are performed for each condition to ensure the statistical reliability of the results. Under these conditions, only the snapshot number is altered to investigate its influence on DOA estimation accuracy.

**Figure 9:**
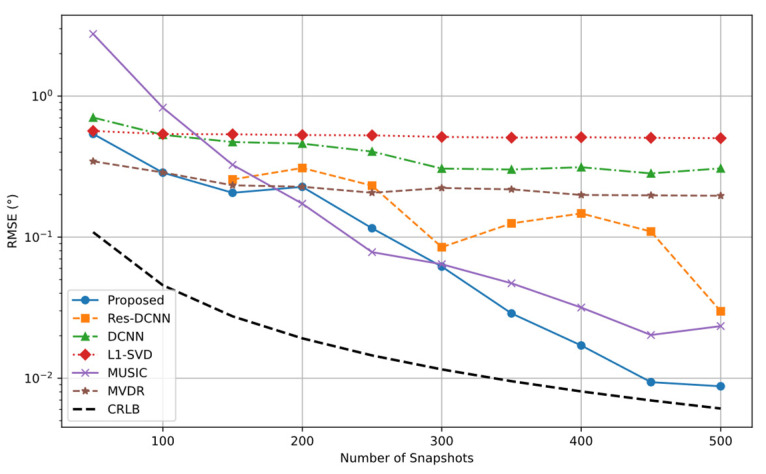
RMSE curves of different number of snapshots for various methods.

As shown in Figure 9, the number of snapshots has a significant impact on the DOA estimation accuracy of each method. Overall, most methods exhibit a decreasing trend in RMSE as the number of snapshots increases. However, noticeable differences in performance are observed among the methods under both low and high snapshot conditions. For snapshot numbers below 150, the MUSIC method produces notably higher RMSE values than other methods. While the MVDR method shows better performance than MUSIC, its overall error remains considerable, and the RMSE reduction with increasing snapshots is marginal. The L1-SVD approach exhibits relatively stable RMSE values over the entire snapshot range, consistently higher than those of other methods. In contrast, the DCNN and Res-DCNN methods show a reduction in RMSE as the snapshot number increases, but the decrease is gradual, and after *T* > 300, the convergence rate slows, and even slightly rebounds. Under *T* < 150, the RMSE of the proposed method is slightly higher than that of the MVDR method. As the number of snapshots increases, the error decreases more noticeably. When the snapshot count exceeds 200, the RMSE becomes significantly lower than that of other methods and gradually approaches the CRLB, demonstrating the effectiveness of the proposed method in different scenarios.

### 4.5. Resolution Probability Analysis for Closely Spaced Sources

In order to assess the capability of the model in handling closely spaced signal sources, the resolution probability is defined as follows: In each simulation, let ${\hat{\theta }}_{1}$ and ${\hat{\theta }}_{2}$ be the estimated angles corresponding to the true angles $\theta_{1}$ and $\theta_{2}$. If the sum of the absolute differences $\left|{\hat{\theta }}_{1}-\theta_{1}\right|+\left|{\hat{\theta }}_{2}-\theta_{2}\right|\leq 2{}^{\circ}$, we consider that simulation a successful resolution; otherwise, it is considered a resolution failure, and the resolution probability is obtained by computing the ratio of successful resolution occurrences. Two groups of experiments are configured to examine the effects of SNR and the number of snapshots, with the signal source angles uniformly set at $\theta_{1}=-1.32{}^{\circ}$ and $\theta_{2}=2.57{}^{\circ}$. The first group of experiments involves varying the SNR from −10 dB to 10 dB in 2 dB steps, with a fixed snapshot number of *T* = 256. In the second group, the number of snapshots is varied from 50 to 500 in steps of 50, while the SNR is held constant at 5 dB. For each SNR or snapshot condition, 600 Monte Carlo simulations are carried out to ensure result reliability.

**Figure 10:**
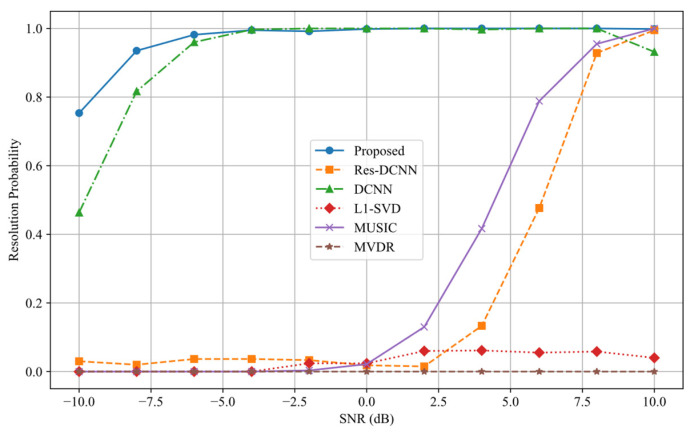
Resolution probability curves of different SNR for various methods.

**Figure 11:**
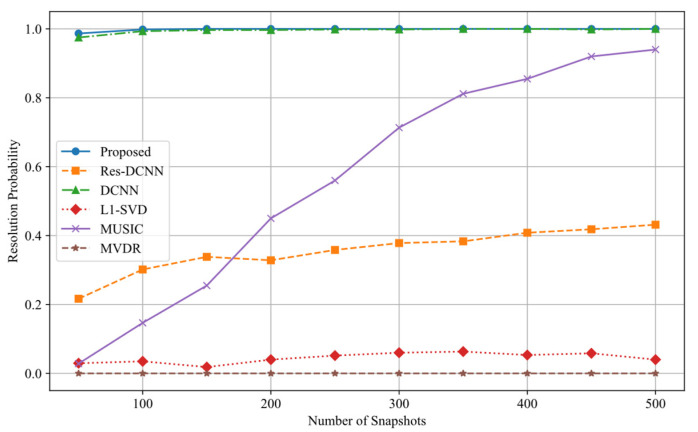
Resolution probability curves of different number of snapshots for various methods.

The experimental results shown in Figure 10 and Figure 11 indicate that both the SNR and the number of snapshots have a significant impact on the ability of each method to resolve closely spaced signal sources. (It should be noted that the L1-SVD and MVDR methods fail to effectively distinguish adjacent signal sources under the current angular separation settings and are therefore excluded from further analysis.) Figure 10 clearly indicates that the proposed method delivers the best performance throughout the full SNR range. It retains the ability to distinguish closely spaced sources even when SNR < −5 dB, demonstrating excellent noise robustness. At 0 dB SNR, the resolution probability approaches 100%, which is significantly higher than that of other methods, confirming its effectiveness in low-SNR conditions. In low SNR scenarios (SNR < 0 dB), the Res-DCNN and MUSIC methods exhibit an inability to effectively resolve signal sources. Although the DCNN method demonstrates a relatively high resolution probability at SNR > –5 dB, its overall performance remains inferior to the proposed method when considering the results of all preceding experiments.

Figure 11 shows that the proposed method maintains high resolution capability even under low snapshot conditions, with the resolution probability rapidly approaching 100% as the number of snapshots increases. Conversely, Res-DCNN exhibits consistently weak resolution ability, and while MUSIC shows improvement with more snapshots, its convergence rate is slow and does not match the performance of the proposed method.

### 4.6. Computational Efficiency Evaluation

**Figure 12:**
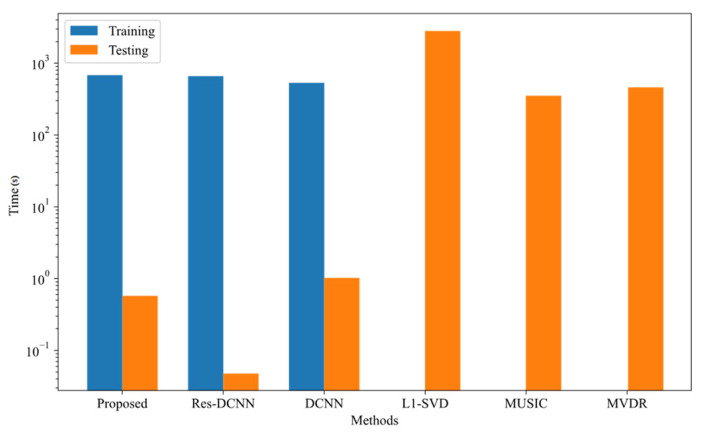
Execution time comparison for various methods.

The computational complexity of each method is evaluated by presenting their respective time metrics in Figure 12 and Table 1. We constructed 10,000 random test samples distributed over different angles and SNR ranges, and all other parameters were set in accordance with Section 4.1. Experiments were conducted under Python 3.9 using PyTorch version 1.11.0 (with CUDA 11.3) on an NVIDIA GeForce RTX 4060 Ti GPU with 32 GB RAM. All randomly generated samples were processed using different methods, and the total prediction time was recorded. Compared to the other two DL-based approaches, the proposed model incurs a longer training time owing to its more complex architecture, which allows for direct input-to-angle estimation mapping. Although conventional methods bypass the need for training, experimental results show that DL models, once trained, can compute DOA estimates efficiently even with moderate computational resources. Due to its structural complexity, the proposed model exhibits marginally longer testing times than other DL models, yet remains significantly faster than traditional techniques, offering real-time capability alongside high measurement accuracy.

## 5. Conclusions

In this paper, we propose an innovative and robust DL-based framework for DOA estimation, featuring a CRDCNN-LSTM network integrated with an enhanced FD loss function. By combining cross-residual convolutional layers with LSTM units, the CRDCNN-LSTM architecture effectively captures both spatial and temporal signal characteristics, thereby enhancing the capability for signal feature extraction. The designed FD loss function, combining Focal Loss and Dice Loss, enhances model focus on weak and low-confidence signals and enforces sparsity in the output spectrum, leading to improved estimation accuracy and robustness under low SNR and limited snapshot conditions. Additionally, applying peak detection and quadratic interpolation in the post-processing stage further refines DOA predictions by mitigating discretization errors. Simulation results demonstrate that, without requiring prior knowledge of the number of sources, the proposed method achieves substantial RMSE reduction under various SNR and snapshot conditions, while offering higher resolution probability and greater computational efficiency compared to conventional algorithms. Moreover, relative to existing DL approaches, it also achieves lower RMSE, higher resolution probability, and notably stronger generalization capability. These quantitative results confirm the significant advantages of the proposed algorithm. Future work will focus on integrating this method into joint communication and sensing systems for real-world applications.

## Figures and Tables

**Table 1:** Time consumption comparison across different DOA estimation methods.

Time(s)	Proposed	Res-DCNN	DCNN	L1-SVD	MUSIC	MVDR
**Training**	690.0886	667.3496	540.3342	/	/	/
**Testing**	0.5806	0.0480	1.0333	2837.47	356.6572	466.0343

## Data Availability

The original contributions presented in the study are included in the article; further inquiries can be directed to the corresponding author.
